# Treatment of humeral shaft fractures with different treatment methods: a network meta-analysis of randomized controlled trials

**DOI:** 10.1186/s12891-023-06626-0

**Published:** 2023-07-17

**Authors:** Hao Qiu, Yuting Liu, Yu Chen, Zheng Weng, Dun Liu, Jing Dong, Minpeng Lu

**Affiliations:** 1 Trauma Orthopedics and Hand Foot Ankle Surgery, The Ninth People’s Hospital of Chongqing, Chongqing, 400700 China; 2 Department of Endocrinology, The Ninth People’s Hospital of Chongqing, Chongqing, 400700 China; 3 Department of Clinical Medicine, Chongqing Medical and Pharmaceutical College, Chongqing, 401331 China; 4 Department of Pain Medicine, the First Affiliated Hospital of Chongqing Medical University, Chongqing, 400016 China

**Keywords:** Non-operative, Open reduction and plate osteosynthesis, Minimally invasive plate osteosynthesis, Intramedullary nail, Humeral shaft fracture, Network meta-analysis

## Abstract

**Purpose:**

Humeral shaft fractures (HSFs) can be treated non-operatively (Non-OP), with open reduction and plate osteosynthesis (ORPO), minimally invasive plate osteosynthesis (MIPO), or with intramedullary nails (IMN). However, the best treatment for HSFs still remains controversial.We performed a network meta-analysis to explore which should be the best method for HSFs.

**Methods:**

The computerized search had been conducted on electronic databases PubMed, EMBASE, Cochrane Library, and Medline from the establishment of the database to the end of December 2022. The quality evaluation of the included literature had been completed by Review Manager (version 5.4.1). Stata 17.0 software (Stata Corporation, College Station, Texas, USA)was used for network meta-analysis.We included randomized controlled trials (RCTs) comparing different treatments to treating HSFs.

**Results:**

The pairwise comparison results demonstrated that there was no statistical difference between IMN, MIPO, Non-OP, and ORPO in terms of radial nerve injury and infection, and Non-OP presented significantly more nonunion than ORPO, IMN, and MIPO. However, no statistically significant difference between ORPO, IMN, and MIPO was discovered. The results of the network meta-analysis displayed that surface under the cumulative ranking curve (SUCRA) probabilities of IMN, MIPO, Non-OP, and ORPO in radial nerve injury were 46.5%, 66.9%, 77.3%, and 9.3%, respectively, in contrast, that in infection were 68.6%, 53.3%, 62.4%, and 15.4%, respectively, and that in nonunion were 51.7%, 93.1%, 0.7%, and 54.5%, respectively.

**Conclusion:**

We came to the conclusion that MIPO is currently the most effective way to treat HSFs.

**Trial registration:**

Name of the registry: Prospero, 2. Unique Identifying number or registration ID: CRD42023411293.

## Introduction

Humeral shaft fractures(HSFs) are common injuries, constituting 1% to 5% of all fractures in adults [1, 2], andthe treatment of HSFs includes operative and non-operative(Non-OP) treatment [3]. This disease was treated conservatively, with a functional Brace, because they [4–7] were regarded as being able to heal with high rates of union and satisfied patients. Many academics, however, concur that surgery has better results [8–10]. Among them, the most common surgical methods are open reduction and plate osteosynthesis (ORPO), minimally invasive plate osteosynthesis(MIPO), and intramedullary nail (IMN) [8].

Non-operative treatment has the advantages of no surgical risk, low treatment cost, and no wound infection [7], but it also has disadvantages, such as nonunion and dysfunction [11]. Traditional ORPO can perform the anatomical reduction of fractures under direct vision, but it also has disadvantages, such as large surgical trauma, wound infection, and nonunion due to excessive periosteal stripping [12]. Minimally invasive plate osteosynthesis can better protect the blood supply of the broken end, minimize the peeling of the periosteum, and reduce surgical trauma, but it also has disadvantages, such as being more difficult to reposition during operation [13]. Intramedullary nail can protect the integrity of the periosteum and the blood supply of fracture ends, and promote fracture healing, but it also has disadvantages, such as poor anti-rotation ability and shoulder impact [14, 15]. Because each of the four different treatment methods has advantages and disadvantages, there is still controversy about the best treatment for HSFs.

There are many meta-analyses to compare the advantages and disadvantages of four different treatment methods for HSFs [11, 16–18]. There are also some network meta-analyses [19, 20] to compare the effects of different surgical methods on HSFs. However, there is no network meta-analysis comparing these four different treatment methods.

Therefore, we use the method of network meta-analysis to evaluate the clinical efficacy of different treatment methods for HSFs, and to provide evidence-based medical evidence for clinical practice.

## Materials and methods

### Search methods

The computerized search had been conducted on electronic databases PubMed, EMBASE, Cochrane Library, and Medline from the establishment of the database to the end of December 2022, according to the PRISMA (Preferred Reporting Items for Systematic Reviews and Meta-Analyses), for RCTs comparing different treatments in the treatment of HSFs. The following keywords and their respective combinations were used:"HSFs", "non-operative", "plate", "intramedullary nail", "open reduction and plate osteosynthesis", "minimally invasive osteosynthesis", "randomized controlled trials", and "randomized". The references of pertinent documents were searched in an effort to increase recall rates.

### Selection criteria

Selection criteria: (1) patients with HSFs aged over 15 years; (2) interventions were Non-OP, ORPO, IMN, and MIPO; (3) comparisons between any 2 of the 4 methods were included; (4) RCTs.

Exclusion criteria: (1) retrospective studies or case reports; (2) full text not available; (3) therewere no outcomes of interest inthereport.

### Quality assessment

The two evaluators (Qiu H and Liu YT) independently screened the literature according to the inclusion and exclusion criteria by reading the title, abstract, and full text of the literature, and discussing and resolving the differences or soliciting the opinions of a third party(Chen Y). The Cochrane Risk of Bias Tool of Review Manager version 5.4 (Copenhagen, Denmark: The Nordic Cochrane Centre, The Cochrane Collaboration) was used to evaluate the quality of the included literature. We evaluate random sequence generation, allocation concealment, blinding of participants and personnel, blinding of outcome assessment, incomplete outcome data, selective reporting, and other biases. Each of these factors was recorded as low risk, unclear risk, or high risk. Where data were unclear, we contacted authors for clarification, where possible. Disagreements were resolved by third-party adjudication.

### Data extraction

Two researchers (Qiu H and Liu YT) independently read the title, abstract and full text of the literature to determine whether the literature met the inclusion criteria and extract data. Extracted information included the first author, publication year, country, study design, characteristics of participants (such as age, and gender), outcome indicators, and information to assess the risk of bias.

### Outcome

Primary outcomes were radial nerve injury, infection, and nonunion.

### Pairwise meta-analysis and network meta-analysis

The quality evaluation of the included literature is completed by Review Manager (version 5.4.1). We use Stata 17.0 software (Stata Corporation, College Station, Texas, USA) for pairwise meta-analysis and network meta-analysis. We used the risk ratio (RR) with 95% confidence intervals (CIs) to calculate the dichotomous outcomes. Display results using the surface under the cumulative ranking curve (SUCRA). The SUCRA value is the percentage under the curve, with a range of 0%-100%, 100% indicates the best treatment, and 0% is the worst.

### Inconsistency analysis

The divergence between direct evidence and indirect evidence indicates that the transitivity hypothesis may not be tenable. We compared the posterior mean deviance contributions of individual data points with the consistency and inconsistency model and node splitting analysis. *P* > 0.05 or 95% CI of inconsistent factors including the null value indicated no significant inconsistency. Inconsistency analysis is shown as a funnel plot.

## Results

### Search results

**Fig. 1:**
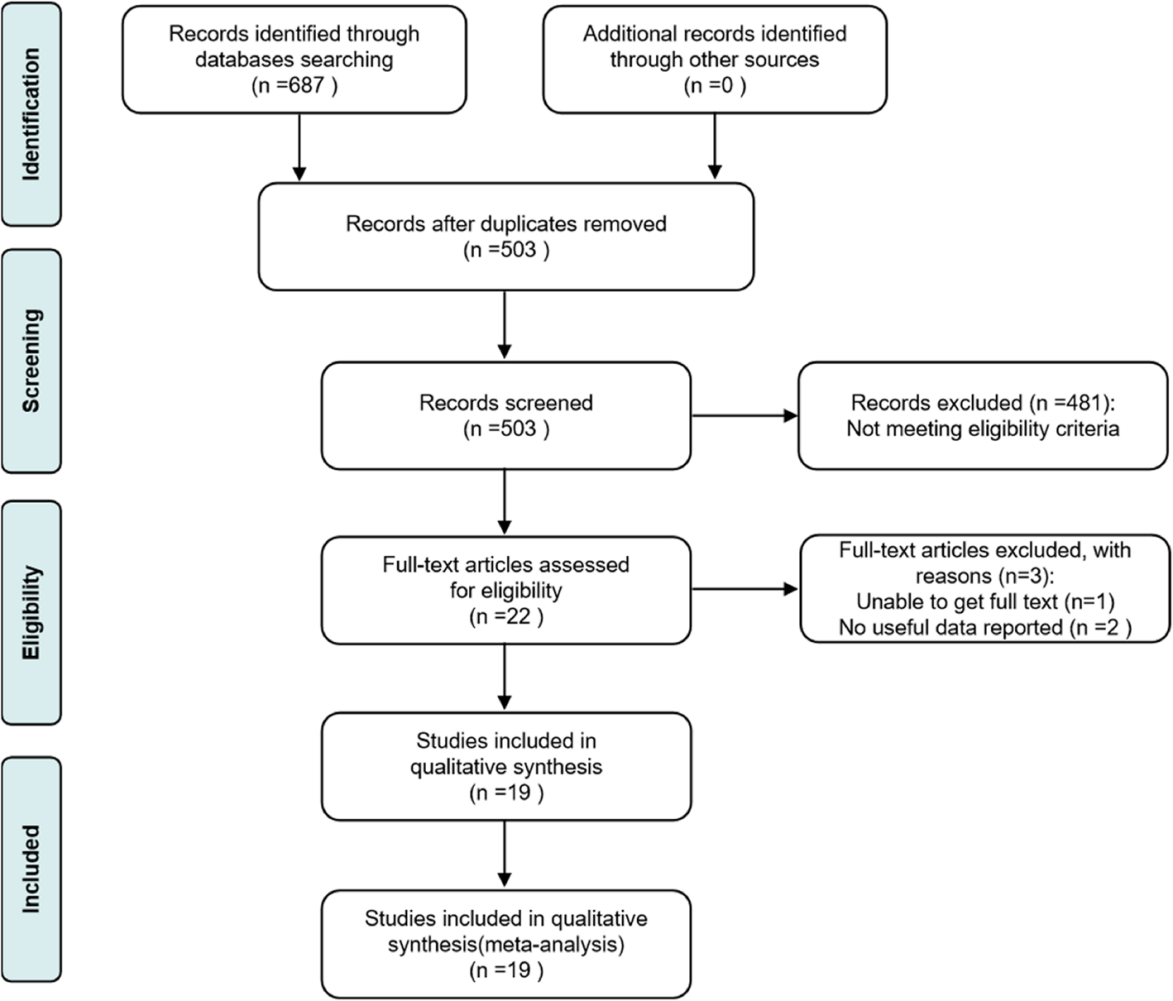
PRISMA 2009 flow diagram. PRISMA: Preferred Reporting Items for Systematic Reviews and Meta-Analyses

**Fig. 2:**
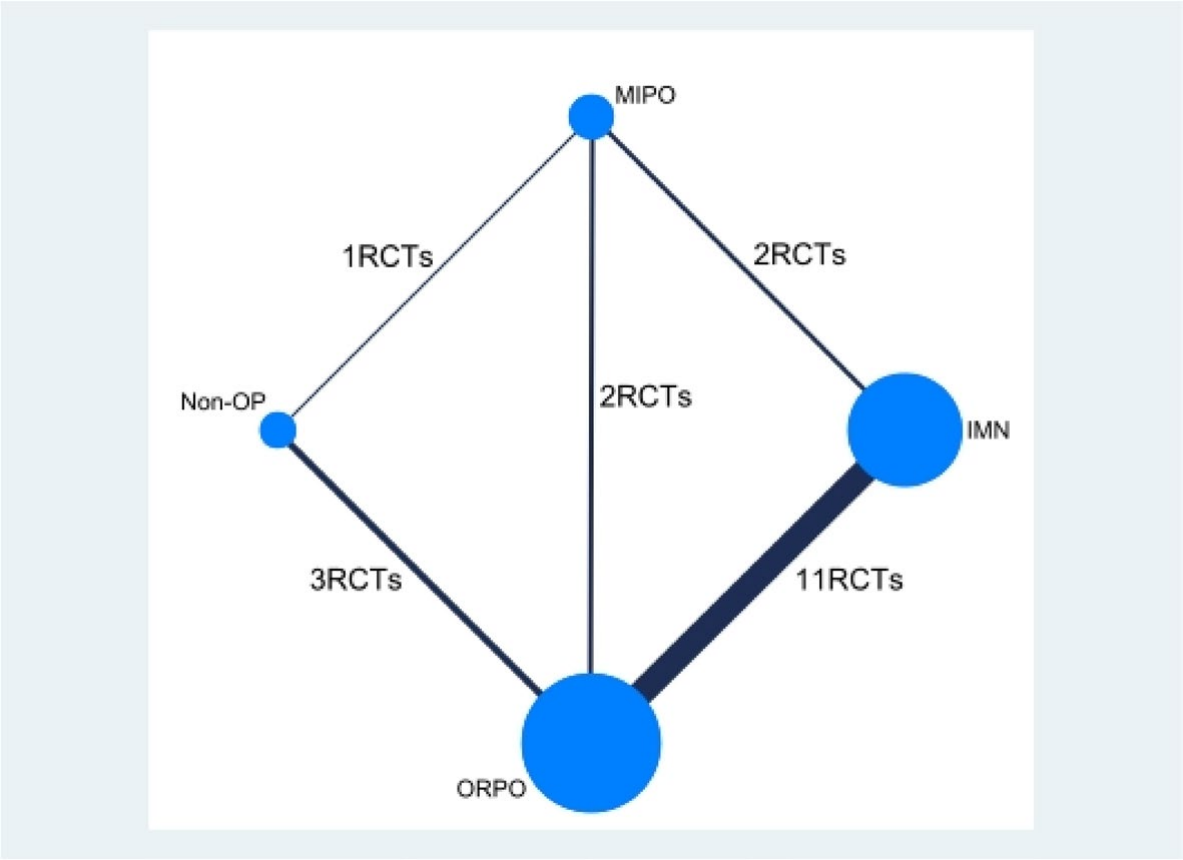
Network diagram between interventions in the network meta-analysis

Out of the 687 records screened from the database, we removed duplicate records and preliminarily screened 22 [21–42] records that met the inclusion criteria by reading the entire text. One RCT [42] was excluded because we could not obtain the full text. Two RCTs [37, 40] were excluded because they did not report outcomes of interest. Finally, our network meta-analysis selected 19 RCTs [21–36, 38, 39, 41]. The study selection process and elimination reasons are shown in Fig. 1. The network diagram between interventions in the network meta-analysis is shown in Fig. 2.

### Quality assessment and basic information

**Fig. 3:**
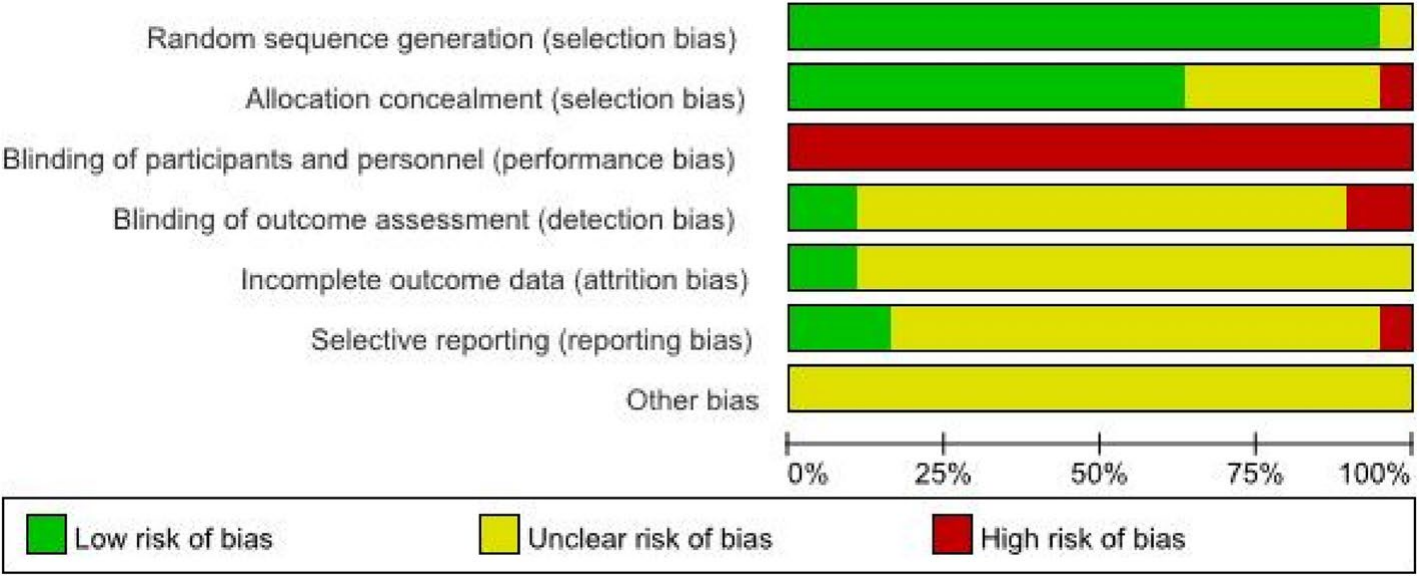
Risk of bias graph

**Fig. 4:**
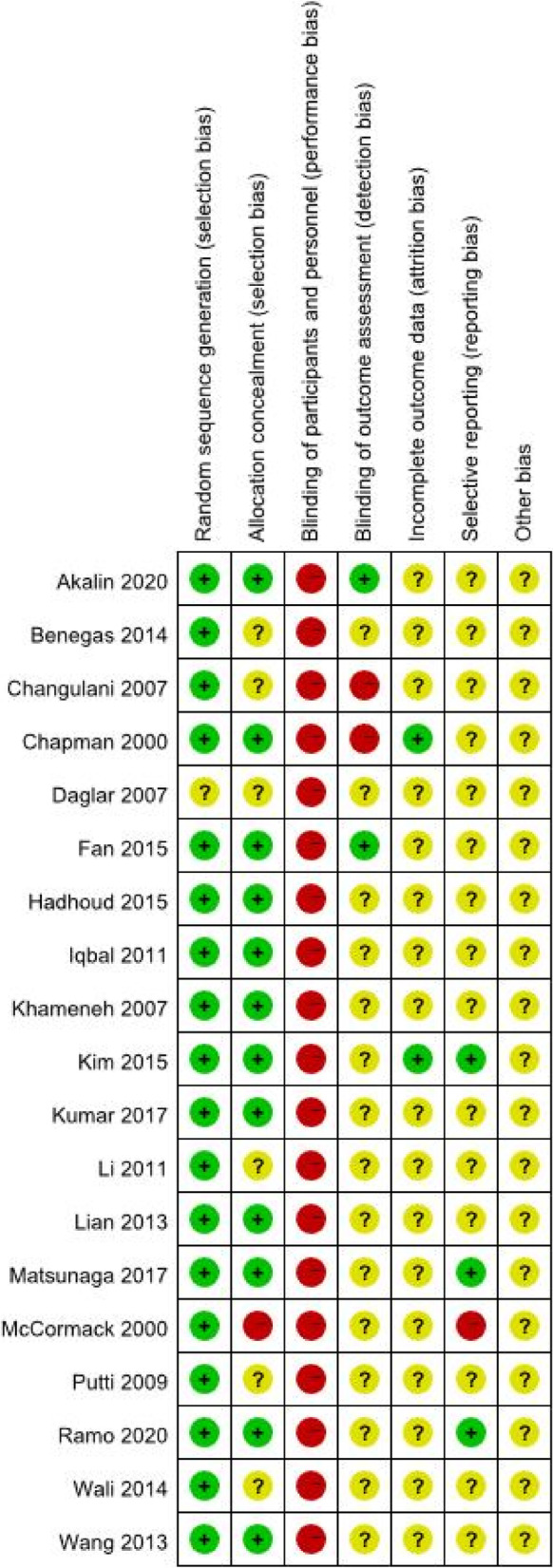
Risk of bias summary

The quality of the included RCTs was assessed using the Cochrane Collaboration’s "Risk of bias". The risk of bias assessment of included studies is given in Fig. 3 and Fig. 4. 19RCTs [21–36, 38, 39, 41] were included, and the characteristics of the included studies are shown in Table 1. These studies were published between 2000 and 2020. All the studies had two eligible arms.

**Table 1:** Characteristics of the included studies

Study (year)	Country	Study type	M/F	Age	Comparison	Main outcome	Follow-up
Chapman 2000 [24]	USA	RCT	51/33	33 (18–83)	ORPO vs. IMN	①②③⑥	13
McCormack 2000 [32]	Canada	RCT	28/16	44.5 (19–82)	ORPO vs. IMN	①②③⑦	14.3
Changulani 2007 [23]	India, UK	RCT	39/8	37	ORPO vs. IMN	①②③⑤⑦	12
Daglar 2007 [25]	Turkey	RCT	14/20	36.4 (18–62)	ORPO vs. IMN	①②③④⑧	32
Wang 2013 [36]	China	RCT	32/13	37.6(20–60)	ORPO vs. IMN	①②③⑦	18
Putti 2009 [33]	India, UK	RCT	32/2	36 (23–84)	ORPO vs. IMN	①②③⑦	24
Akalin 2020 [21]	Turkey	RCT	41/22	43.25(18–88)	ORPO vs. IMN	①②③④⑤⑦⑨	12
Iqbal 2011	Pakistan	RCT	30/10	28 (15–40)	ORPO vs. IMN	①④	12
Li 2011	China	RCT	35/15	37.6 (20–60)	ORPO vs. IMN	①③⑥⑧	12
Wail 2014	India	RCT	41/9	37.5	ORPO vs. IMN	①②③④	12
Fan 2015 [26]	China	RCT	37/23	39.25	ORPO vs. IMN	①②④⑤⑦⑧	12
Lian 2013 [30]	China	RCT	31/16	38.2 (17–77)	IMN vs. MIPO	①②③④⑤⑥⑦	14.5
Benegas 2014 [22]	Brazil	RCT	26/14	41.6	IMN vs. MIPO	①②③⑥⑨	12
Kim 2015 [28]	Korea	RCT	37/31	42 (15–86)	ORPO vs. MIPO	①②④⑤⑥⑨	15
Hadhoud 2015 [39]	Egypt	RCT	20/10	37 (20–67)	ORPO vs. MIPO	①②③④⑤⑨	10
Khameneh 2019 [27]	Iran	RCT	49/11	43.1(18–77)	Non-OP vs. ORPO	①②③⑤⑨	12
Kumar 2017 [38]	India	RCT	29/11	35.18(18–83)	Non-OP vs. ORPO	②⑤⑥	6
Ramo 2020 [34]	Finland	RCT	44/38	49(19–81)	Non-OP vs. ORPO	①②③⑧	12
Matsunaga 2017 [31]	Brazil	RCT	73/37	38.8	Non-OP vs. MIPO	①②③⑧	12

*RCT* randomized controlled trial *M* male, *F* female, *Non-OP* Nonoperative, *ORPO* open reduction and plate osteosynthesis, *IMN* intramedullary nailing, *MIPO* minimally invasive plate osteosynthesis, *vs*. versus; ① Radial nerve injury; ② Nonunion; ③ Infection; ④ Operation time; ⑤ Union time; ⑥ Malunion; ⑦ American Shoulder and Elbow Surgeons score; ⑧ Constant score; ⑨ the University of California, Los Angeles score

### Results of network meta-analysis

#### Radial nerve injury

**Fig. 5:**
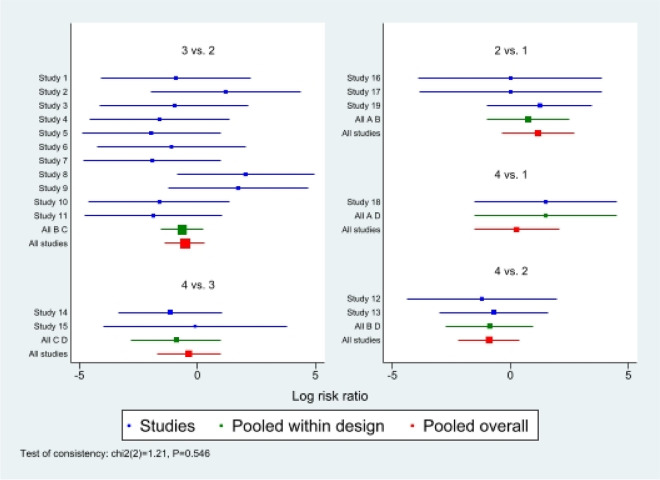
Inconsistency of the included studies. 1: Nonoperative (Non-OP); 2: open reduction and plate osteosynthesis(ORPO); 3:intramedullary nailing (IMN);4:minimally invasive plate osteosynthesis (MIPO)

**Fig. 6:**
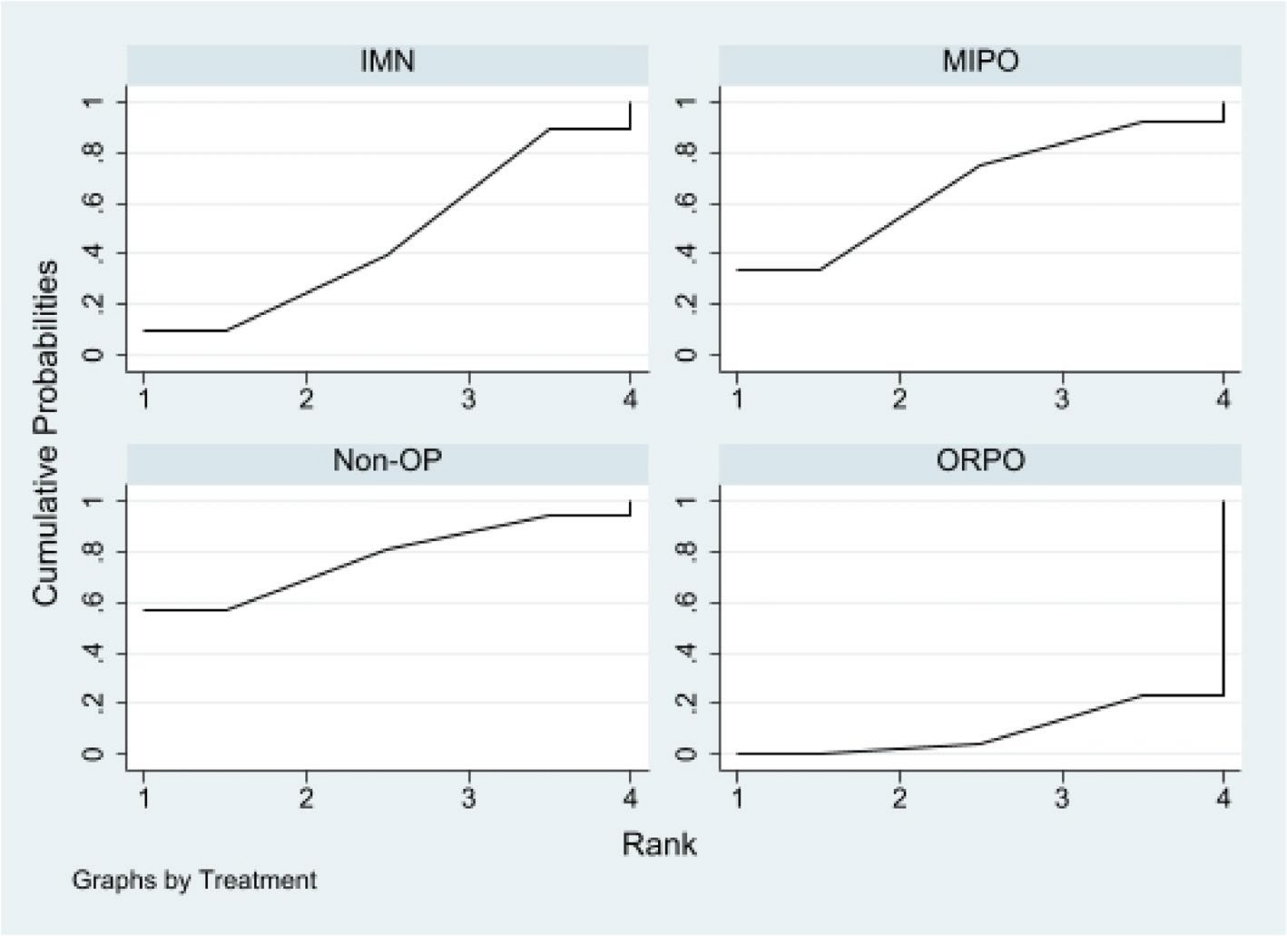
The surface under the cumulative ranking curve for radial nerve injury. Non-OP: Nonoperative; ORPO: open reduction and plate osteosynthesis; IMN: intramedullary nailing; MIPO: minimally invasive plate osteosynthesis

**Fig. 7:**
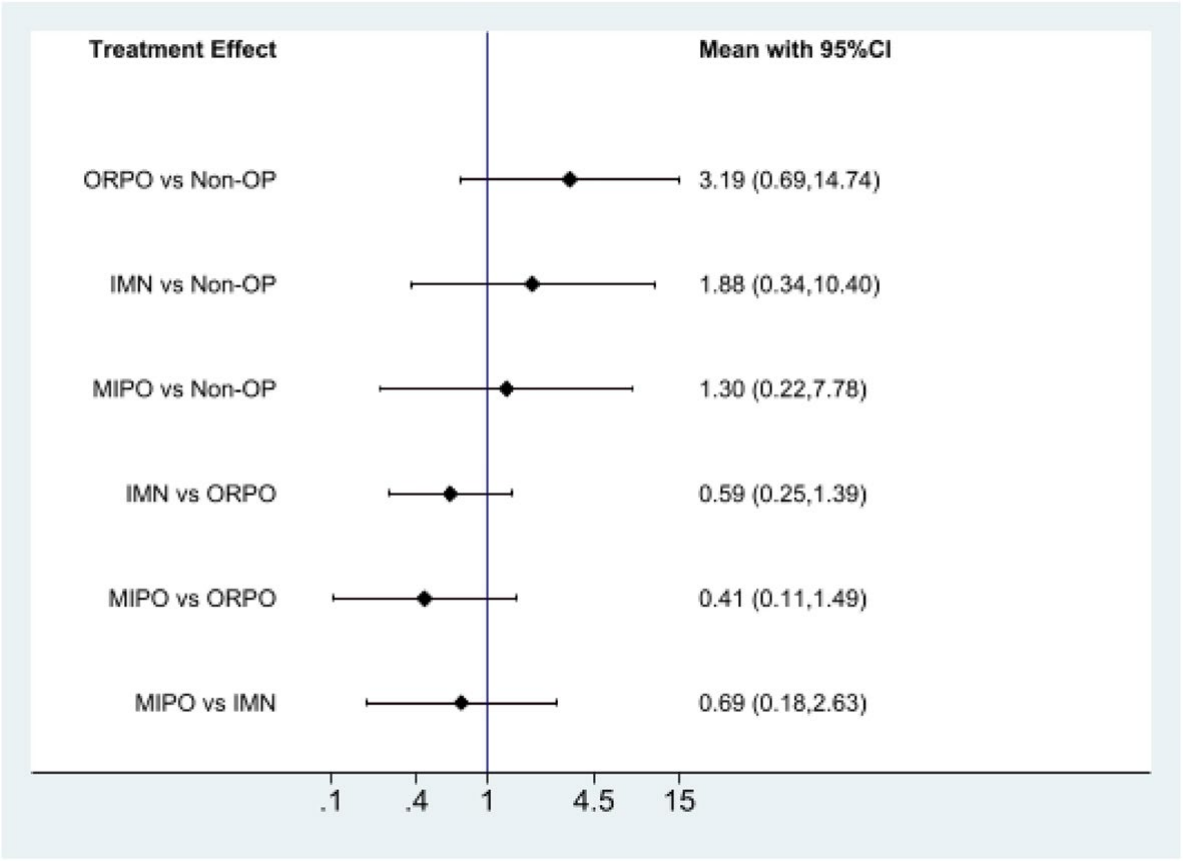
The pairwise comparison of the included studies. Non-OP: Nonoperative; ORPO: open reduction and plate osteosynthesis; IMN: intramedullary nailing; MIPO: minimally invasive plate osteosynthesis

First of all, we analyzed the global inconsistency of the included literature. The results showed that there was no inconsistency in the included literature (*P* = 0.546)(Fig. 5). The results of the network meta-analysis showed that SUCRA probabilities were 46.5%, 66.9%, 77.3%and 9.3% for IMN, MIPO, Non-OP, and ORPO, respectively (Fig. 6). The pairwise comparison results show that no statistical difference was found between IMN, MIPO, Non-OP, and ORPO (*P* > 0.05) (Fig. 7).

#### Infection

**Fig. 8:**
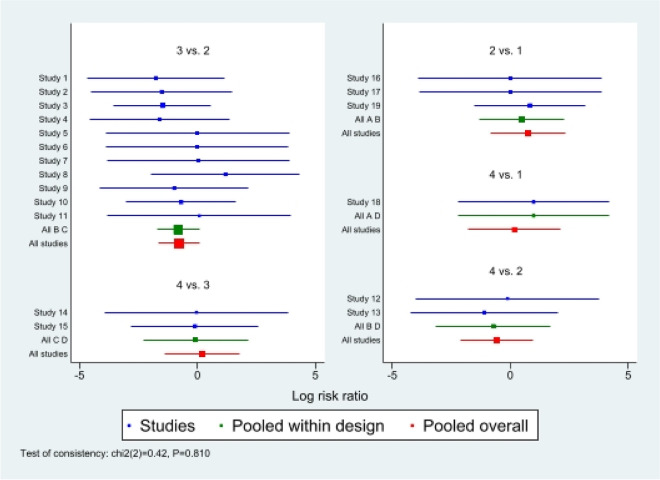
Inconsistency of the included studies. 1: Nonoperative(Non-OP); 2:open reduction and plate osteosynthesis (ORPO); 3:intramedullary nailing (IMN);4:minimally invasive plate osteosynthesis (MIPO)

**Fig. 9:**
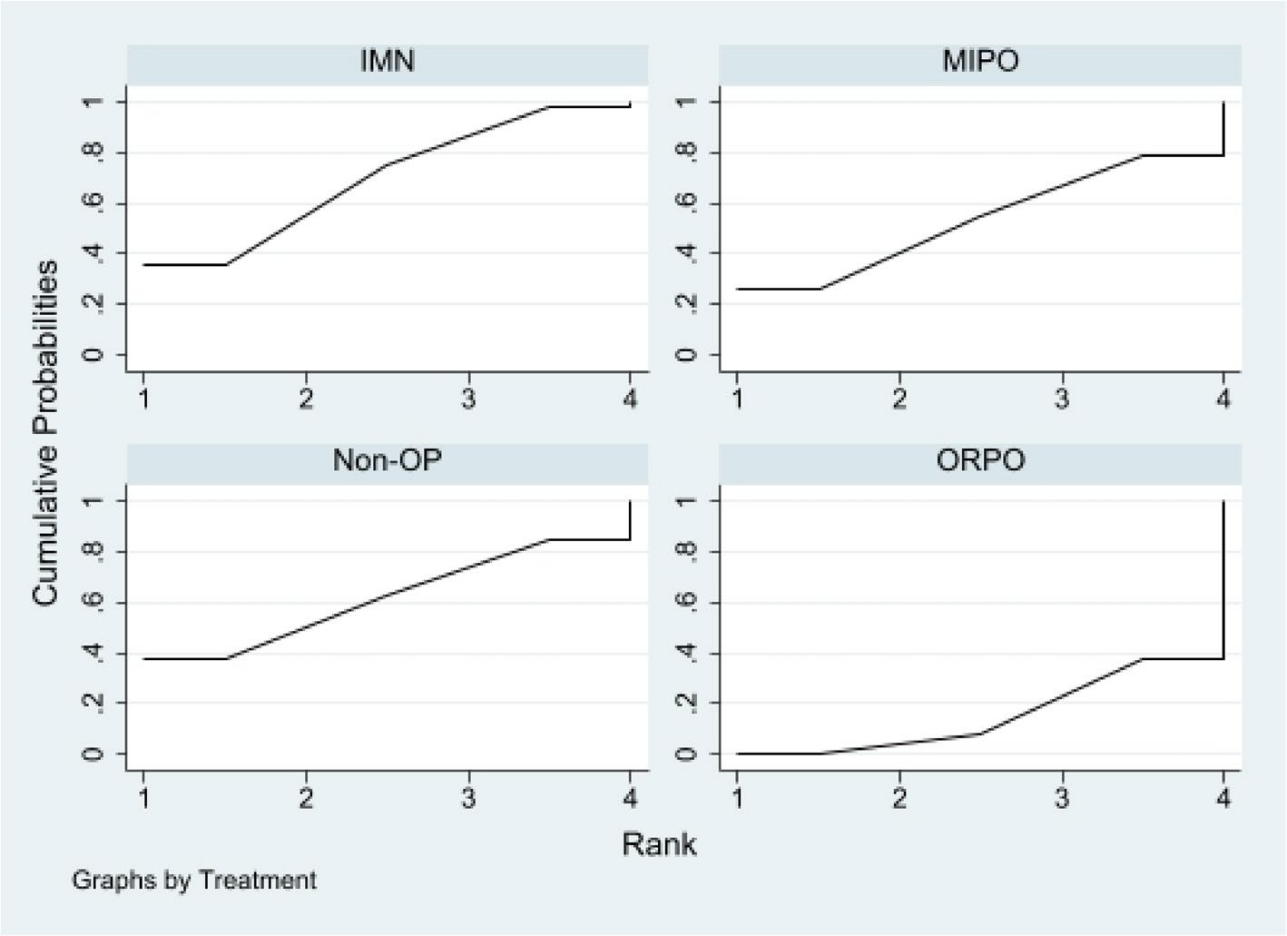
The surface under the cumulative ranking curve for infection. Non-OP: Nonoperative; ORPO: open reduction and plate osteosynthesis; IMN: intramedullary nailing; MIPO: minimally invasive plate osteosynthesis

**Fig. 10:**
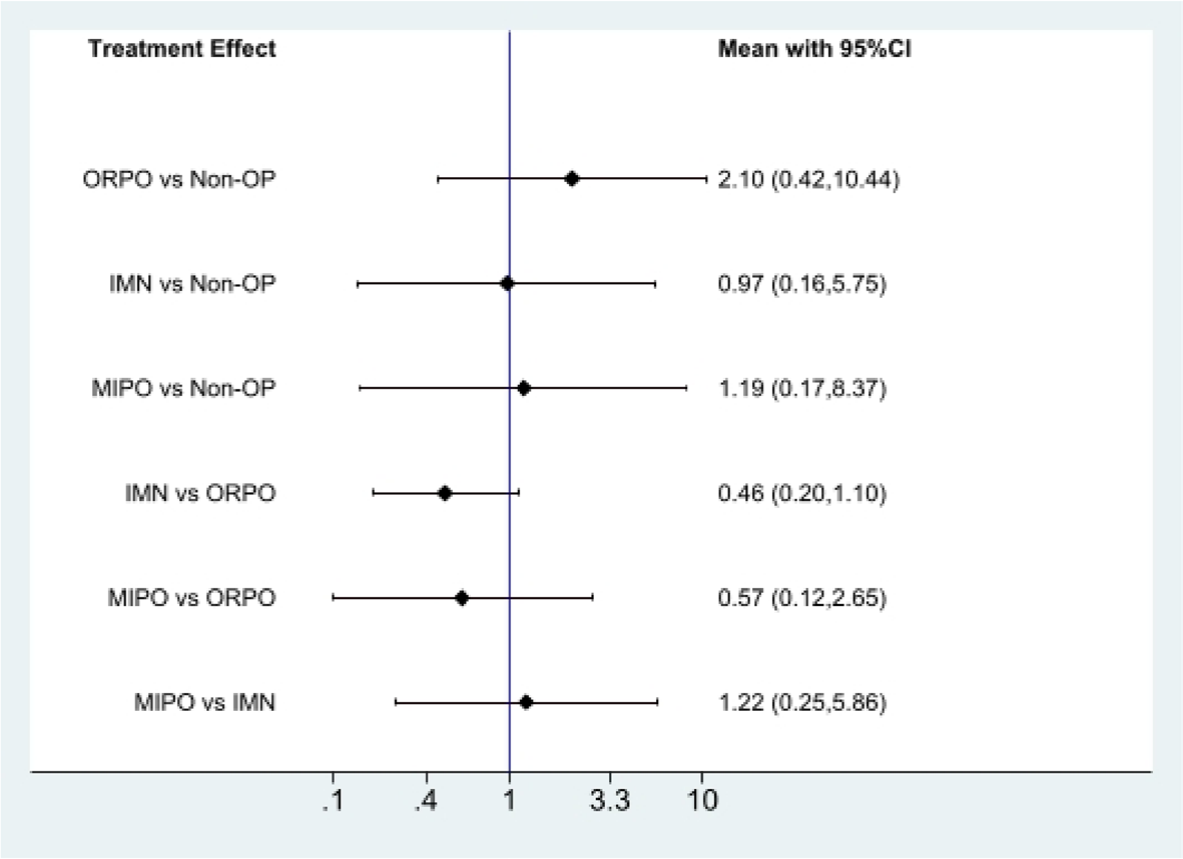
The pairwise comparison of the included studies. Non-OP: Nonoperative; ORPO: open reduction and plate osteosynthesis; IMN: intramedullary nailing; MIPO: minimally invasive plate osteosynthesis

First of all, we analyzed the global inconsistency of the included literature. The results showed that there was no inconsistency in the included literature (*P* = 0.810) (Fig. 8). The results of the network meta-analysis showed that SUCRA probabilities were 68.6%, 53.3%, 62.4%and 15.4% for IMN, MIPO, Non-OP, and ORPO, respectively (Fig. 9). The pairwise comparison results show that no statistical difference was found between IMN, MIPO, Non-OP, and ORPO (*P* > 0.05)(Fig. 10).

#### Nonunion

**Fig. 11:**
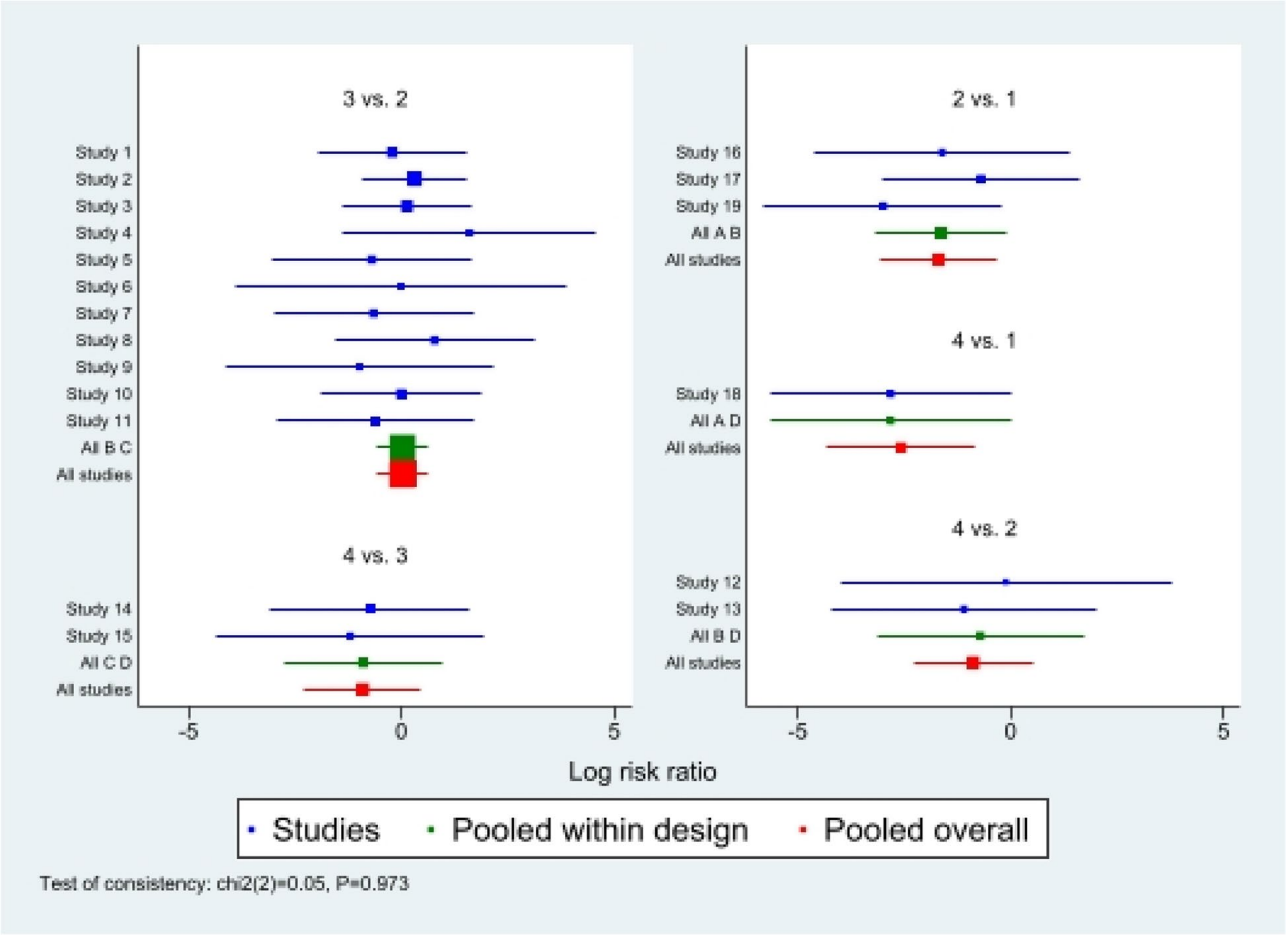
Inconsistency of the included studies. 1: Nonoperative (Non-OP); 2:open reduction and plate osteosynthesis(ORPO);3:intramedullary nailing (IMN);4:minimally invasive plate osteosynthesis (MIPO)

**Fig. 12:**
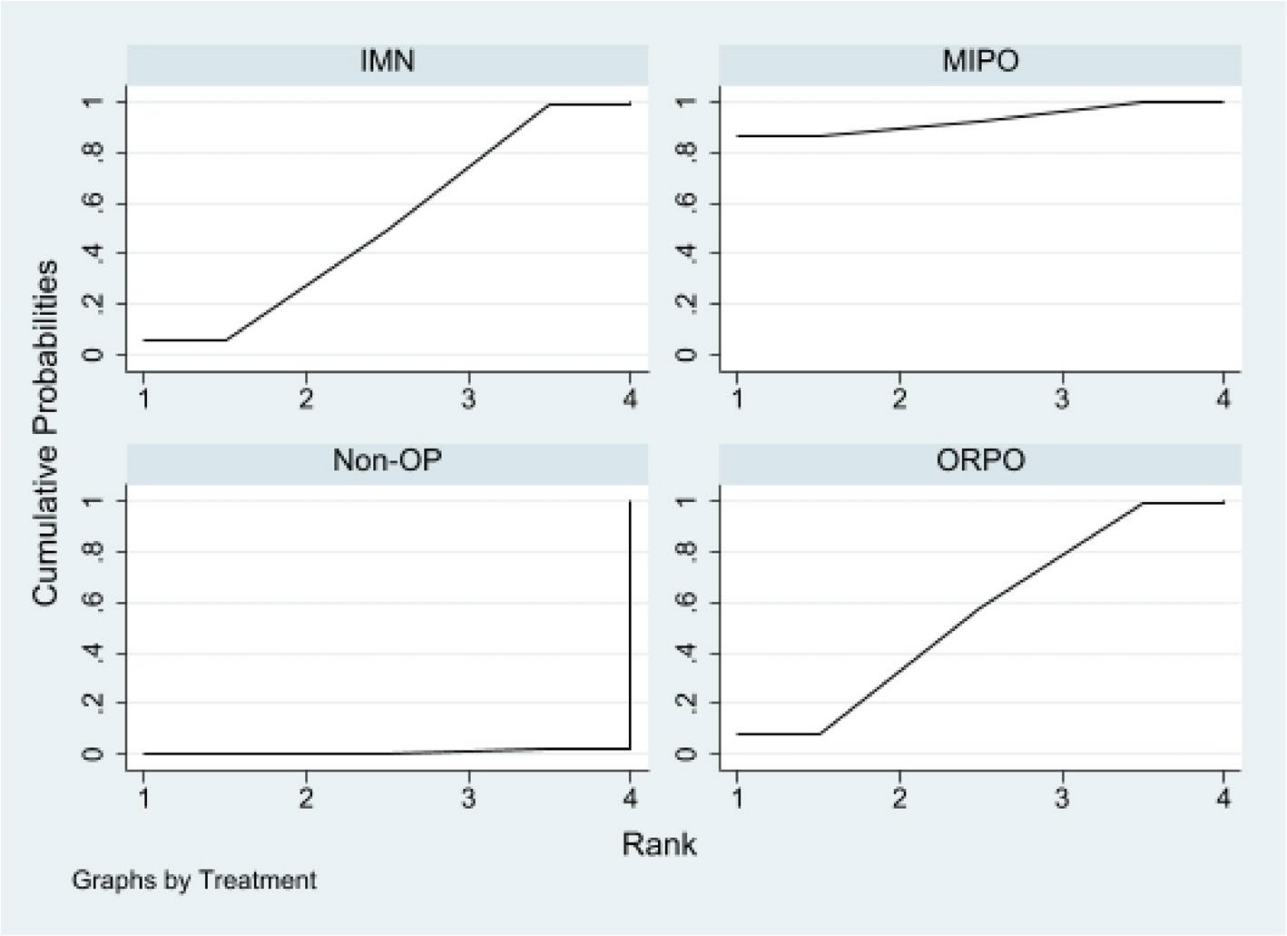
The surface under the cumulative ranking curve for nonunion. Non-OP: Nonoperative; ORPO: open reduction and plate osteosynthesis; IMN: intramedullary nailing; MIPO: minimally invasive plate osteosynthesis

**Fig. 13:**
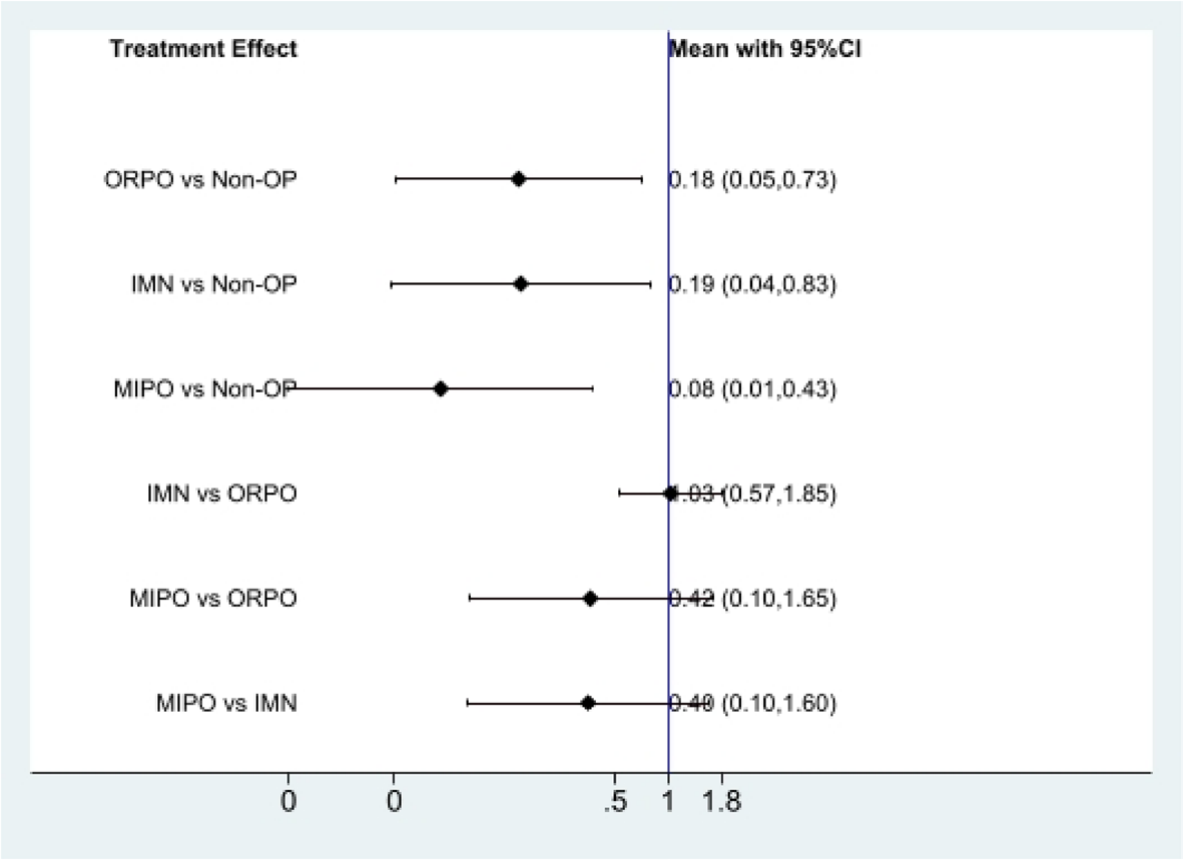
The pairwise comparison of the included studies. Non-OP: Nonoperative; ORPO: open reduction and plate osteosynthesis; IMN: intramedullary nailing; MIPO: minimally invasive plate osteosynthesis

First of all, we analyzed the global inconsistency of the included literature. The results showed that there was no inconsistency in the included literature (*P* = 0.973) (Fig. 11). The results of the network meta-analysis showed that SUCRA probabilities were 51.7%, 93.1%, 0.7%and 54.5% for IMN, MIPO, Non-OP, and ORPO, respectively (Fig. 12). The pairwise comparison results proved that Non-OP presented significantly more nonunion than ORPO [RR: 0.18, 95% CI: 0.05 to 0.73, *P* < 0.05], IMN [RR: 0.19, 95% CI: 0.04 to 0.83, *P* < 0.05], MIPO [RR: 0.08, 95% CI: 0.01 to 0.43, *P* < 0.05].There was no statistically significant difference between ORPO, IMN, and MIPO (*P* > 0.05) (Fig. 13).

#### Publication bias and inconsistency analysis

**Fig. 14:**
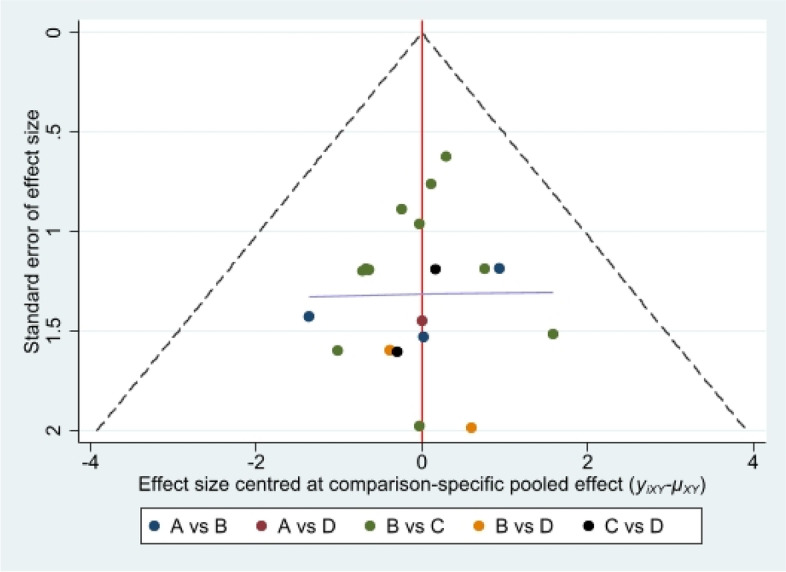
Funnel plot of the network meta-analysis. **A**: Nonoperative (Non-OP); **B**: open reduction and plate osteosynthesis(ORPO); **C**:intramedullary nailing(IMN); **D**:minimally invasive plate osteosynthesis (MIPO)

**Fig. 15:**
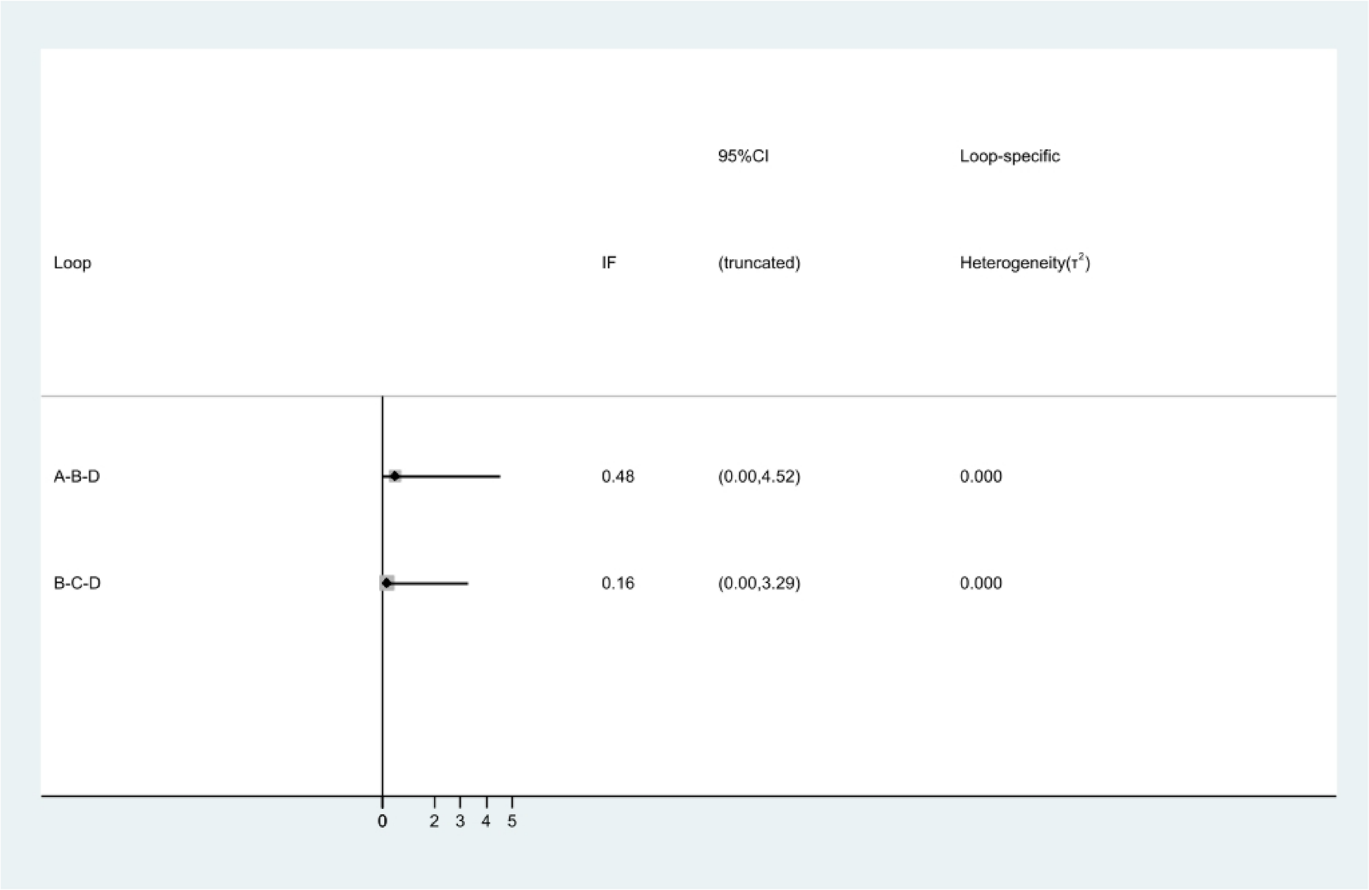
Plot for identifying inconsistency in network meta-analysis

In general, the funnel plot was symmetrical, indicating a slight publication bias (Fig. 14). The null value was included in the 95% CI for the inconsistency analysis (Fig. 15), demonstrating that all direct and indirect evidence is consistent and there is no inconsistent evidence in the network meta-analysis.

## Discussion

The treatment method of HSFs has been mainly divided into operative treatment and non-operative treatment [3], and the most common surgical methods involve open reduction and plate osteosynthesis (ORPO), minimally invasive plate osteosynthesis (MIPO), and intramedullary nail (IMN) [8]. At present, the best treatment for HSFs is still up for debate [5, 9, 43, 44]. Moreover, there has been not enough evidence of a direct comparison between non-operative and intramedullary nails. For that reason, we consider it necessary to conduct an updated network meta-analysis to compare the impact of all four methods on HSFs.

The pairwise comparison results showcased that there was no statistical difference between IMN, MIPO, Non-OP, and ORPO in terms of radial nerve injury and infection. In spite of that, the results of the SUCRA ranking prove that the probability of radial nerve injury in Non-OP is the lowest than that in MIPO, ORPO, and IMN, and the probability of infection in IMN is the lowest than that in MIPO, ORPO, and Non-OP. The pairwise comparison results display that Non-OP presented significantly more nonunion than ORPO, IMN, and MIPO. Though, there was no statistical difference was found between ORPO, IMN, and MIPO. Additionally, according to the results of the SUCRA ranking, MIPO has the lowest probability of nonunion compared with Non-OP, ORPO, and IMN, while Non-OP has the highest probability compared with ORPO and IMN.

Also, we discovered that ORPO significantly scored lower on the SUCRA scale for radial nerve injury compared with the other three groups, while MIPO and Non-OP scored similarly. IMN, MIPO, and Non-OP all have similar SUCRA rankings for infection, whereas ORPO's was significantly lower than the other three groups. In terms of nonunion, Non-OP had a significantly lower SUCRA ranking for nonunion than the other three groups, while MIPO had a significantly higher SUCRA ranking than the other two groups. Thus, we believe that although the probability of radial nerve injury and infection in Non-OP is low, the probability of nonunion is high. MIPO has a higher probability of bone healing and a lower probability of infection and radial nerve injury. Intramedullary nail has a low probability of infection, but its probability of non-union and radial nerve injury is relatively high. In ORPO, there is a high risk of infection and radial nerve injury, as well as a moderately high probability of non-union. Subsequently, according to the results of our network meta-analysis, we think MIPO is the best method to treat HSFs at present.

The function of the elbow and shoulder after HSFs must be taken into account. We did not assess the shoulder and elbow joint function because the included study's shoulder and elbow joint function evaluation indicators were inconsistent. Kumar S [38] showed that the functional outcome after operative treatment was better than the non-operative treatment. The meta-analysis findings of van de Wall BJM [18]proved that the recovery of shoulder and elbow joint function in MIPO is better than that in IMN. The meta-analysis results of Hu Y [17] unveiled that the recovery of shoulder and elbow joint function in ORPO is better than that in IMN. The meta-analysis results of Beeres FJ [16] exhibited that there is no difference between ORPO and MIPO in functional recovery of shoulder and elbow joints. Consequently, we infer that ORPO and MIPO are superior to IMN and Non-OP in functional recovery of shoulder and elbow joints, while ORPO and MIPO have similar functional recovery of shoulder and elbow joints.

Still, our network meta-analysis has potential limitations. First, due to the evaluation indicators and data types included in the study are not entirely consistent, the data that we can combine and analyze is not sufficient, such as the shoulder joint and elbow joint function score. Second, the inclusion study adopts various inclusion and exclusion criteria and follow-up times, causing the heterogeneity observed in the trial. Third, we only analyzed the effect of different treatment methods on postoperative complications of HSFs but did not analyze its impact on functional recovery and daily life. Fourth, it can be seen from the network diagram that there are few RCTs between some treatments. If more randomized controlled trials can be included in the future, a more convincing result can be obtained.

## Conclusion

We thought the Non-OP treatment is more likely to result in bone nonunion, while ORPO and MIPO are superior to IMN and Non-OP in functional recovery of shoulder and elbow joints. Nevertheless, compared with MIPO, ORPO is prone to develop complications such as radial nerve injury and infection.Therefore, we deduced that MIPO is currently the most effective way to treat HSFs. Many high-quality RCTs are still required in order to further confirm the aforementioned findings in the future because our network meta-analysis only included a small number of studies.

## Data Availability

All data generated or analysed during this study are included in this publishedarticle.
